# Liraglutide Enhances the Activity of the ACE-2/Ang(1–7)/Mas Receptor Pathway in Lungs of Male Pups from Food-Restricted Mothers and Prevents the Reduction of SP-A

**DOI:** 10.1155/2018/6920620

**Published:** 2018-12-02

**Authors:** J. Fandiño, A. A. Vaz, L. Toba, M. Romaní-Pérez, L. González-Matías, F. Mallo, Y. Diz-Chaves

**Affiliations:** Laboratory Endocrinology, LabEndo, Centro de Investigaciones Biomédicas (CINBIO), University of Vigo, E36310 Vigo, Spain

## Abstract

In utero growth restriction and being born small for gestational age are risk factors for respiratory morbidity. IUGR (in utero growth retardation) is associated to overall reduction in lung weight, surfactant content and activity, impaired maturation of the alveolar type II cells, and decreased alveolar formation. The renin-angiotensin system (RAS) may be a key target underlying pathophysiological lung alterations. GLP-1 and agonists of its receptor modulate the expression levels of different components of RAS and also are very important for lung maturation and the production of surfactant proteins. The aim of this study was to elucidate the effects of IUGR induced by perinatal food restriction of the mother in the lung function of pups at early stages of life (PD21) and to determine if liraglutide had any effect during gestational period. Sprague-Dawley pregnant rats were randomly assigned to 50% food restriction (MPFR) or ad libitum control (CT) groups at day of pregnancy 12 (GD12). From GD14 to parturition, pregnant MPFR and CT rats were treated with liraglutide or vehicle. At postnatal day 21 and before weaning, 20 CT and 20 FR male pups were sacrificed and lungs were analyzed by RT-PCR. Liraglutide restored surfactant protein A (SP-A) mRNA expression in pup lungs from food-restricted mothers. Surfactant protein B (SP-B) mRNA expression is not affected by neither IUGR nor liraglutide treatment. Moreover, liraglutide modulated different elements of RAS, increasing angiotensin-converting enzyme 2 (ACE2) and MasR mRNA expression only in pups from food-restricted mothers (MPFR), despite food restriction had not any direct effect at this early stage. Liraglutide also increased endothelial nitric oxide synthase (eNOS) expression in MPFR lungs, reflecting the activation of MasR by angiotensin 1–7. In conclusion, liraglutide prevented the alteration in lung function induced by IUGR and promoted the positive effects of ACE2-Ang(1–7)-MasR in restoring lung function.

## 1. Introduction

Lung development in mammals is initiated at embryogenesis (in rats begins about day 13, 13E); it continues through fetal development, and it is completed at postnatal life, from P7 to P21 in rats [1]. During fetal development, the cellular proliferation leads to the ramification pattern of the respiratory tree and drives the differentiation of many of cells including pulmonary epithelium. The type two (II) pneumocytes are the epithelial cells responsible of the synthesis and secretion of pulmonary surfactant, which consist in phospholipids (90%) and proteins (10%). The surfactant reduces surface tension in the interphase of the pulmonary alveoli preventing their collapse [2]. There are two types of surfactant proteins (SPs): hydrophilic SPs (SP-A and SP-D) and hydrophobic SPs (SP-B and SP-C) [3]. SP-A is the most abundant SP, and it maintains the pulmonary surfactant physicochemical stability and contributes to the in situ innate immune response [4]. SP-B is essential to respiratory function and when absent limits survival shortly after birth [5]. The appropriate fetal pulmonary surfactant production is necessary for extrauterine life, and so any event affecting the production of surfactant proteins may delay lung maturation and compromise the postnatal respiratory function [6].

In humans, low weight at birth is a risk factor associated to impaired pulmonary function in the perinatal period [7] and in the adulthood [8]. Factors affecting the intrauterine environment, such as maternal malnutrition, reduce fetal growth and lungs' size and maturity [9]. In fact, maternal malnutrition is one of the most impacting conditions to induce intrauterine growth retardation (IUGR; [10, 11]). In sheep fetuses submitted to placental restriction and thus showing IUGR, there is a marked reduction in the expression of mRNA for SP-A, SP-B, and SP-C [12]. Moreover, IUGR increases the risk to suffering respiratory distress syndrome and bronchopulmonary dysplasia at birth [13] with long-term consequences at adulthood [14]. In addition, it has been described a reduced postnatal alveolar formation in a rat model of caloric restriction that mimics IUGR [15]. This fact has been associated with an increase in apoptosis and a diminution of cellular proliferation due to deficient prenatal and postnatal nutrition delivery.

Many studies support that the local action of the renin-angiotensin system (RAS) is involved in the development of the lung [16]. The angiotensin-converting enzyme (ACE) expressed at endothelium of pulmonary vessels cleaves circulating angiotensin I into angiotensin II (AngII) which promotes proliferation and cellular differentiation through type 1 angiotensin receptor (AT1-R). *In vitro* studies revealed that AngII promotes lung branching through AT1-R [16]. AngII also induces antiproliferative effects by the activation of the type 2 angiotensin receptor (AT2-R). In addition, AngII is metabolized to angiotensin 1–7 (Ang(1–7)) by the angiotensin-converting enzyme 2 (ACE2). Ang(1–7) works as an AT1-R antagonist and has antiproliferative actions by competitive counteracting AngII [17]. But Ang(1–7) also binds specifically to another receptor, the Mas receptor. The ACE-2/Ang(1–7)/Mas receptor axis usually has opposite actions to AngII/AT1R activation and then promotes vasodilation [18]. All components of RAS are highly expressed in fetal lungs [16] supporting a key role of these molecules in the lung development, as previously described the implications of AngII in kidney, heart, and liver organogenesis [19].

GLP-1 is a gastrointestinal peptide having pleiotropic effects in insulin secretion [20], hypothalamic control of food intake [21], regulation of the hypothalamic-pituitary-adrenal [22–24] and gonadotrophic axis [25], modulation of gastrointestinal motility [26], and regulation of lipid metabolism [27] among others. Glucagon-like peptide-1 receptor (GLP-1R) is also widely expressed in lungs [28–30], where it is more abundant than in any other tissues [31]. Interestingly, the GLP-1R is expressed at different stages of fetal lung development showing a very marked increase just the day after birth, afterwards maintained during adulthood [32]. In addition, the GLP-1R natural ligand, the native GLP-1 (1–36)-NH_2_, is particularly important for the production of surfactant proteins, SP-A and SP-B, contributing to pulmonary development and function [32]. We have recently proven that liraglutide, a potent agonist of the GLP-1 receptor, is able to potently modulate the pulmonary expression levels of the RAS components including ACE and ACE2, in adult rats [30]. Despite the large amount of data summarized above, the putative role of GLP-1R agonists in respiratory diseases has not been studied in deep up to date.

The aim of this study was to elucidate the effects of IUGR in an animal model induced by maternal food restriction, in the lung expression of surfactant proteins A and B and ACEs and AT receptors, and to determine if liraglutide (and analogue of GLP-1 receptor) during gestational period may have any effect.

## 2. Material and Methods

Female Sprague-Dawley rats (8 weeks old) supplied by the University of Santiago de Compostela (Spain) were housed in the animal facility of the University of Vigo (Spain), in a 12/12 h of light/dark cycle, controlled temperature conditions (20–22°C) and ad libitum access to standard feed (A04-Panlab, Barcelona, Spain) and tap water, unless otherwise stated. Special care was taken to minimize suffering and to reduce the number of animals used to the minimum required for statistical accuracy. The experimental procedures were conducted under the European Union guidelines for the use of animals for experimental purposes (Council Directive 2010/63/EU) and had been approved by the ethical committee of the University of Vigo and Xunta de Galicia (ES360570215601/17/FUN01/FIS02/LCGM/02).

The estrous cycle of female rats was daily monitored by vaginal cytology. The females were housed with males for mating during 24 hours at proestrous. The day of vaginal plug was considered as day 1 of gestation (GD1). At GD12, pregnant rats were then randomly assigned to food restriction (50% of daily intake of control dams. MPFR; *n* = 10) or ad libitum control groups (CT; *n* = 10) and individually housed in plastic breeding cages. From GD14 to parturition, both groups (MPFR and CT) were treated with liraglutide (100 *μ*g/kg s.c./twice a day; CT/LIR and MPFR/LIR, *n* = 6; Bachem, Bubendorf) or with vehicle (saline and acetic acid 0.4%, s.c.; MPFR/VEH or CT/VEH). At parturition, litter size was adjusted to eleven pups per dam, with similar numbers of males and females. After delivery, mothers from the MPFR group were leave to a feeding regimen of 70% of the daily food consumed by the ad libitum rats during lactation. At postnatal day 21 (PD21) and before weaning, 40 males (20 controls and 20 MPFR of each group) were sacrificed by decapitation, trunk blood was collected, and the lower lobe of the left lung was removed and immediately frozen until processed for RT-PCR. To obviate any litter effects, animals used for each experiment were randomly chosen from different litters and only a limited number of animals (*n* = 1–2) was used from each litter. Male animals were preferred in this study since clinical and experimental data indicate that lung development under structural and functional aspects is accelerated in female compared with male preterm and term neonates, which may underlie a lower perinatal and late neonatal mortality in females [33, 34] and make males more suitable for the study of lung experimental pathology [34].

### 2.1. Real-Time PCR Analysis

Surfactant proteins A (SP-A) and B (SP-B), Mas receptor (MasR), cell surface angiotensin II receptor 1 (ATR-1) and 2 (ATR-2), angiotensin-converting enzyme 1 (ACE1) and 2 (ACE2), endothelial nitric oxide synthase (eNOS), inducible nitric oxide synthase (iNOS), *β*-actin, and GAPDH as housekeeping mRNA levels were assessed in male lungs by using TRI Reagent® solution following the manufacturer's instructions (Ambion, USA). Primer sequences were designed using Primer Blast (NCBI; Table 1). First-strand cDNA was prepared from 2 *μ*g RNA from the caudal lobe of lungs using RevertAid Reverse Transcriptase (Thermo Scientific, Massachusetts, United States) according to the manufacturer's protocol. The cDNA was amplified in duplicate by real-time PCR using a SYBR Green master mix in an Agilent Technologies 7900HT ABI Prism 7500 Sequence Detector (AB), with conventional AB cycling parameters (40 cycles of 95°C, 15 s; 60°C, 1 min). Data are represented using the comparative cycle threshold (Ct) method. To validate a ΔΔCt value, we checked that the amplification efficiency of the target and the reference genes was comparable (the absolute value of the slope of ΔCt vs. log relative concentration between −0.1 and 0.1). The Ct was determined for each target and reference gene in duplicate. Then, ΔΔCt was calculated by normalizing the ΔCt of each sample to the mean ΔCt value of the male CT/VEH group.

### 2.2. Statistical Analysis

Data are presented as mean ± SEM. Statistical analyses were performed using GraphPad Prism6 software (GraphPad Software, San Diego, CA, USA). Main and interactive effects were analyzed by one-way ANOVA, followed by its Tukey post hoc multiple comparison test. Differences were statistically significant at *p* ≤ 0.05.

## 3. Results

### 3.1. Effect of Maternal Perinatal Food Restriction (MPFR) in Pup's Body Weight

**Figure 1:**
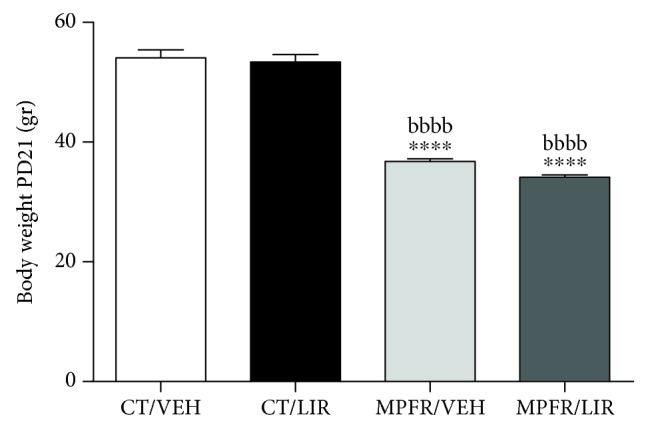


Male pups from perinatal food-restricted mothers displayed intrauterine growth retardation as reflected by their significantly lower body weight (*n* = 8; 36.8 ± 0.43 grams) compared to pups from control rats (*n* = 8; 54.08 ± 1.36 grams) or controls treated with liraglutide (*n* = 8; 53.37 ± 1.23 grams; Figure 1; one-way ANOVA, *F*_(3,24)_ = 86.83, *p* < 0.0001; MPFR/VEH vs. CT/VEH *p* < 0.0001; MPFR/VEH vs. CT/LIRA *p* < 0.0001; Tukey's multiple comparison test). Administration of liraglutide did not revert body weight decrease of male pups from food-restricted dams (*n* = 4; 38.03 ± 1.86 grams; Figure 1, MPFR/LIRA vs. CT/VEH *p* < 0.0001 and MPFR/LIRA vs. CT/LIRA *p* < 0.0001, ANOVA followed by post hoc Tukey's multiple comparison test).

### 3.2. Liraglutide Administration to Food-Restricted Pregnant Rats Restored Pulmonary SP-A mRNA Expression in Male Pups (PD21)

**Figure 2:**
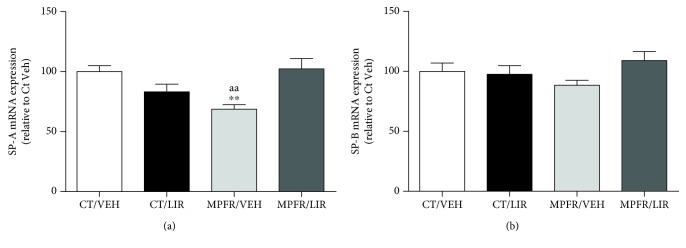


The long-lasting effects of MPFR as well as liraglutide influence on SP-A and SP-B were studied before weaning in PD21 pups. Maternal food restriction (MPFR) decreased SP-A mRNA levels in a 30% compared to controls (MPFR/VEH; *n* = 8; Figure 2(a); one-way ANOVA, *F*_(3,24)_ = 7.67, *p* < 0.0009; MPFR/VEH vs. CT/VEH *p* < 0.01; Tukey's multiple comparison test) without affecting SP-B mRNA expression (Figure 2(b)). Liraglutide administration to ad libitum fed mothers (CT) did not modify pulmonary SP-A or SP-B mRNA levels in lungs from male offspring (*n* = 8; Figures 2(a) and 2(b)). However, the treatment with liraglutide of food-restricted dams (MPFR) was able to restore the SP-A mRNA levels to those of controls (MPFR/LIRA; *n* = 4; Figure 2(a); one-way ANOVA; MPFR/LIRA vs. MPFR/VEH *p* < 0.001; Tukey's multiple comparison test) and has no effect on mRNA SP-B expression (Figure 2(b)).

### 3.3. Effect of Maternal Perinatal Food Restriction (MPFR) and Liraglutide Treatment in RAS Components

**Figure 3:**
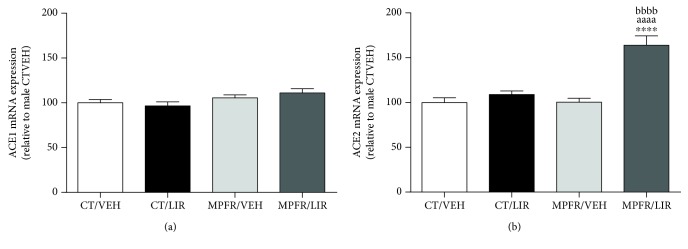


**Figure 4:**
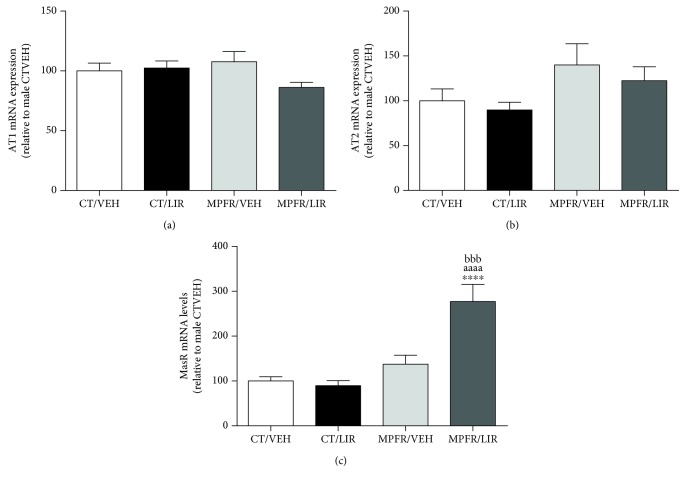


The long-lasting effects of maternal undernutrition as well as of liraglutide administration during gestation in the pulmonary RAS components were analyzed (Figures 3 and 4). The mRNA expression of the angiotensin-converting enzyme 1 and 2 (ACE1 and ACE2) was studied (Figure 3) in the lung of PD21 male pups. MPFR had any effect in the mRNA expression of these enzymes (Figures 3(a) and 3(b)). The liraglutide administration to control dams did not affect ACE1 (Figure 3(a)) or ACE2 (Figure 3(b)) mRNA levels too. However, liraglutide administration to food-restricted mothers (MPFR) increased the mRNA expression in lungs of ACE2 by 1.63-fold, compared to pups from the groups of control (CT) or MPFR treated with vehicle (Figure 3(b); one-way ANOVA, *F*_(3,24)_ = 22.17, *p* < 0.0001; MPFR/LIRA vs. CT/VEH *p* < 0.0001; MPFR/LIRA vs. MPFR/VEH *p* < 0.0001; MPFR/LIRA vs. CT/LIRA *p* < 0.0001; Tukey's multiple comparison test). No effect of liraglutide treatment in food-restricted dams was observed in ACE1 mRNA expression in lungs of pups (Figure 3(a)).

Concerning AngII receptors, MPFR had no any effect on AT1 or AT2 mRNA expression (Figures 4(a) and 4(b)). Liraglutide treatment of control dams did not affect the mRNA levels of AT1-R or AT2-R (Figures 4(a) and 4(b)) in male pups. Neither the treatment with liraglutide of food-restricted mothers has any effect on AT1 nor AT2 mRNA expression (Figures 4(a) and 4(b)).

Mas receptor, which specifically binds Ang(1–7), was also studied in the lungs of PD21 male pups. MPFR did not modify the expression of the Mas receptor; neither the treatment with liraglutide of control dams modifies the mRNA expression of this receptor. However, liraglutide markedly increased the mRNA expression of MasR in the lungs of MPFR pups compared to controls treated with vehicle or liraglutide and from MPFR pups treated with vehicle (Figure 4(c); one-way ANOVA, *F*_(3,23)_ = 17.45, MPFR/LIRA vs. CT/VEH *p* < 0.0001; MPFR/LIRA vs. CT/LIRA *p* < 0.0001; and MPFR/LIRA vs. MPFR/VEH *p* < 0.001, Tukey's multiple comparison test).

### 3.4. Effect of Maternal Perinatal Food Restriction (MPFR) and Liraglutide Treatment in NOS Synthases

**Figure 5:**
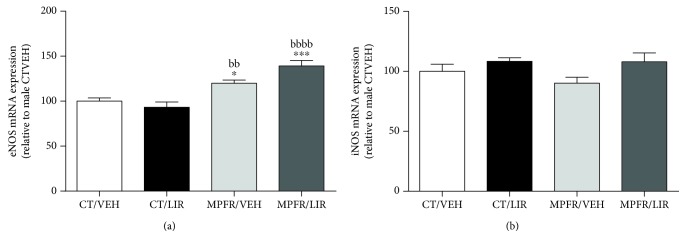


The activation of MasR by Ang(1–7) promotes vasodilatation of the vessel mediated by several mechanisms, including the expression of NOS synthases in the endothelia. We measured the expression levels of eNOS (endothelial synthase) and iNOS (inducible synthase) in the lungs of pups at PD21. MPFR increases the mRNA levels of eNOS in pup lungs compared to controls treated with vehicle or liraglutide (Figure 5(a); one-way ANOVA, *F*_(3,24)_ = 15.2, *p* < 0.0001; MPFR/VEH vs. CT/VEH *p* < 0.05 and MPFR/VEH vs. CT/LIRA *p* < 0.01; Tukey's multiple comparison test) but did not affect iNOS mRNA expression. The treatment with liraglutide of control dams did not affect the mRNA expression of eNOS or iNOS (Figures 5(a) and 5(b)). However, pups from MPFR dams treated with liraglutide showed further increased of the mRNA expression of eNOS compared to controls (Figure 5(a); MPFR/LIRA vs. CT/VEH *p* < 0.001 and MPFR/LIRA vs. CT/LIRA, *p* < 0.0001). No effect of liraglutide on iNOS mRNA expression was observed in MPFR pups (Figure 5(b)).

## 4. Discussion

Most animal models of IUGR have restricted fetal growth because of interference with placental function and uterine blood flow or placental insufficiency. The pathophysiology and phenotype of the abnormalities in lung development resulting from these insults may vary according to species, timing, chronicity, and intensity of the relevant exposure/insult. However, these studies largely confirm that normal lung development is critically dependent on the appropriate oxygen supply and nutrition. IUGR is associated with persisting or developing abnormalities of the structure and function in both the airways and parenchyma [35]. Also in humans, adverse environment in utero can influence lung function in mid adult life. In fact, birth weight is a marker for adult lung function [36].

Pulmonary surfactant was identified as a lipoprotein complex that reduces surface tension at the air-liquid interface of the lung, but surfactant has functions in pulmonary host defense too. More recent studies have shown novel roles for these proteins in the clearance of apoptotic cells, direct killing of microorganisms, and initiation of parturition [37]. Our results showed that the animal model of IUGR induced by maternal perinatal food restriction (MPFR) affected the lung surfactant system in postnatal male rats at 21 days of age, decreasing SP-A mRNA. Moreover, the treatment with liraglutide to dams during pregnancy restored SP-A mRNA expression to those levels observed in control pups. In one study about the fetal-maternal model of food restriction in rats, the authors did not find any change on SP-A or SP-B mRNA levels at age just before weaning [38], likely because unexpectedly the body weight of male pups from food-restricted mothers increased with respect to controls, which may produce some experimental compensatory trend. In another study, in which pups displayed decreased body weight at 28 days of postnatal life due to late pregnancy undernutrition [39], neither observed changes in SP-A nor SP-B mRNA expression at this age. In our model, food restriction of the mothers lasts not just during pregnancy period but also prolongs during the lactation, which seems to be critical to reduce surfactant protein mRNA expression in early stages of postnatal life. SP-A is the most abundant of all surfactant proteins because it is a key factor for the formation of the surfactant monolayer and since even small reductions may affect the interphase monolayer and respiratory mechanics. In addition, SP-A greatly contributes to the innate local immune responses [37], and then, its reduction explains a higher susceptibility to respiratory infections [40].

Local production of angiotensins explains the tissue-specific effects of RAS on growth and differentiation which are thought to be extremely important for embryonic and fetal development [19]. During pulmonary morphogenesis, lung expresses ACE-1 and ACE-2, responsible of local and circulating levels of AngI and AngII and also AT1 and AT2 receptors [41, 42]. Recently, it has been shown that all components of RAS are differentially expressed in rat fetal lungs throughout gestational ages [16]. The RAS function is very important in fetal lung maturation, especially to bronchial-alveolar branching and vascular development, and both processes are stimulated by AngII through the activation of the AT1 receptor [16].

In the uteroplacental unit, RAS interacts with other important systems in order to fine-tune various tissue functions [41]. The Ang(1–7)/ACE2/Mas R axis regulates blood pressure in the pregnant state [42] but also the placental blood flow and its distribution [43]. In addition, ACE-1 expression is reduced in fetal lungs by in utero environmental changes such as the antenatal hypoxia, leading to the upregulation of ACE-2 at both transcriptional and translational levels [44]. Moreover, the in utero environment conditions promote epigenetic changes which modify the expression of several genes of the pulmonary RAS [44].

We have previously shown that GLP-1 receptor agonists are a relevant modulator of the RAS components in the lung, especially stimulating the expression of ACE-2, in different physiological and pathological conditions [30, 32]. Within the RAS, ACE-2 competes with ACE because it is capable of hydrolyzing the inactive decapeptide angiotensin I (AngI) into the nonapeptide Ang(1–9), thus decreasing the amount of Ang I available for pressor AngII generation by ACE. To the same extent, ACE-2 degrades vasoconstrictor AngII into Ang(1–7), which may also be produced from Ang(1–9) hydrolysis by ACE [17]. In the MPFR rats, there are no significant changes in either the expression levels of ACE as was observed in another model of IUGR induced by placental restriction in ewes [45] and food-restricted rats [46] or of ACE-2, in IUGR rats at 4 months of age [46].

However, liraglutide differentially and potently stimulates the levels of ACE-2. In such condition, the local production of Ang(1–7) will greatly increase, activating the MasR. Moreover, liraglutide increased the expression levels of MasR in the lungs of MPFR pups and enhanced the activity of the whole ACE-2/Ang(1–7)/MasR axis in those animals. This axis exerts biological antagonistic actions with respect to the other branches of RAS; the axis conformed by ACE/AII/AT1R [47], and for that, it was also named “vasoprotective axis.” The modulation of the ACE-2/Ang(1–7)/MasR is regarded a novel therapeutic approach to counterbalance the vasoconstrictive, proliferative, and fibrotic actions of ACE/AII/AT1 axis [48]. Moreover, Ang(1–7) reduces lung fibrosis and pulmonary arterial hypertension [49] and activates events that are crucial for the resolution of the inflammatory process of asthma and the promotion of return to lung homeostasis [45]. IUGR or low birth weight was associated with medium and small airway obstruction and altered lung function [50]. It was also associated with increased risk of asthma both in children and adults [51], and IUGR rats are highly sensitive to hypoxia later in life, suffering more significant pulmonary arterial hypertension lung vascular remodeling [52]. Interestingly, liraglutide treatment also increases eNOS expression in the lungs of MPFR pups, which is in congruence with the activation of MasR by Ang(1–7). Receptor Mas stimulation leads to, among others, increased phosphorylation of endothelial nitric oxide synthase (eNOS) and increased nitric oxide (NO) release [53].

It is quite remarkable that all these changes induced by liraglutide in MPFR pups are completely independent of body weight, which is similar in animals treated with liraglutide or vehicle. The altered responses to liraglutide reveal the underlying respiratory dysfunction in MPFR pups, conditioning the increase of the pulmonary pathology in future adult individuals.

Whether the effects induced by liraglutide in RAS function of MPFR pups are a consequence of underlying subclinical alterations in the lungs of these animals or a substantial part of the biological response to improve the lung tissue function should be matter of future studies.

## Figures and Tables

**Table 1:** Primer sequences used for quantitative real-time polymerase chain reaction.

Gene	Forward	Reverse
BACT	CACCATGTACCCAGGCATTG	CCTGCTTGCTGATCCACATC
GAPDH	AGCCCAGAACATCATCCCTG	GCATGTCAGATCCACAACGG
SP-A	CTGCCAGGATTTCCAGCTTAC	TTGACTGACTGCCCATTGGT
SP-B	CTGTGCCAAGAGTGTGAGGA	CAAGCAGCTTCAAGGGTAGG
AT2-R	CCGTGACCAAGTCTTGAAGATG	AGGGAAGCCAGCAAATGATG
AT1-R	TTCGTGGCTTGAGTCCTGTT	GGTGATCACTTTCTGGGAGGG
MasR	CTGGTCAACCTTTGGGAACCT	AAAGGGTTGGCGCTGCTA
ACE1	TCCTGCTAGACATGGAGACGA	CAGCTCTTCCACACCCAAAG
ACE2	CGCTGTCACCAGACAAGAA	CGTCCAATCCTGGTTCAAG
eNOS	TGACCCTCACCGATACAACA	CGGGTGTCTAGATCCATGC
iNOS2	CCCTTCAATGGTTGGTACATG	ACATTGATCTCCGTGACAGCC

## Data Availability

The data used to support the findings of this study are available from the corresponding author upon request.
